# Reply: “Improving the Study of Impulse Control Disorders in CSAI‐Treated Parkinson's Disease: Methodological and Interdisciplinary Directions”

**DOI:** 10.1002/mdc3.70315

**Published:** 2025-09-02

**Authors:** Clément Desjardins, Paulo André Dias Bastos, Margherita Fabbri

**Affiliations:** ^1^ Department of Neurology Rothschild Foundation Hospital Paris France; ^2^ Department of Clinical Pharmacology and Neurosciences Toulouse Parkinson Expert Centre, Toulouse NeuroToul Center of Excellence in Neurodegeneration (COEN), French NS‐Park/F‐CRIN Network, University of Toulouse 3, CHU of Toulouse, INSERM Toulouse France

**Keywords:** continuous subcutaneous apomorphine infusion, impulse control disorders, Parkinson's disease

We thank Wang and colleagues for their thoughtful and constructive comments^1^ on our recent study^2^ examining the effects of continuous subcutaneous apomorphine infusion (CSAI) on impulse control disorders (ICDs) in advanced Parkinson's disease (PD). Our study provides a key hypothesis: CSAI, despite increasing total dopaminergic load, may mitigate ICDs risk—likely via stable dopaminergic stimulation and oral dopamine agonist (DA) reduction. These warrant using targeted, prospective studies. As a large, real‐world cohort study, our work cannot prove causality; however, it provides foundational evidence suggesting that CSAI may be a viable treatment option for advanced PD patients with ICD vulnerability. Larger, controlled trials, ideally incorporating neurobehavioral phenotyping, will be essential to confirm and extend these findings. We appreciate the opportunity to address the points raised and to outline future directions for research in this area.

First, we fully agree that ICDs encompass a heterogeneous group of behaviors, such as gambling, hypersexuality, binge eating, and compulsive shopping, each with specific gender predisposition. However, in real‐life settings, overall severity (as captured by MDS‐UPDRS item 1.6) often guides therapeutic decisions. Our approach reflects this clinical reality and allows detection of consistent attenuation post‐CSAI. Subgroup analyses by ICD subtype or baseline severity, as suggested, are indeed essential for mechanistic insights, but these require large samples and detailed behavioral assessments that are rarely available. For example, in a large cohort study of 3090 PD patients evaluated with dedicated ICD scales, the following subtype‐specific prevalences were reported^3^: gambling 5%, hypersexuality 3.5%, compulsive shopping 5.7%, and binge eating 4.3%, with about 3.9% of patients exhibiting two ICDs. Although certain associations have been described—such as pathological gambling with older age, binge eating with lower anxiety, and hypersexuality or compulsive shopping with higher levodopa doses^3^—few studies have attempted to replicate these findings, likely due to the very large sample sizes required. As a result, ICD risk factors such as DA therapy/dose, younger age, male sex, higher levodopa equivalent daily dose, and family history of ICDs are typically reported at the group level rather than stratified by specific ICD subtype.

Second, as acknowledged in our paper, a limitation of our study is the lack of information on reasons for CSAI drop‐out, which could in theory include ICD worsening. However, prior reviews of CSAI discontinuation^4^, ^5^ have not identified ICDs worsening/appearance as a major reason for treatment withdrawal, making it unlikely that this was a considerable factor in our cohort.

Third, we clearly highlighted that CSAI could be a safe option for patients with slight ICDs as only a real minority of patients has mild‐to‐severe baseline ICDs. This distribution is most likely explained by the selection criteria for CSAI initiation rather than by cohort‐related bias. Nevertheless, the safety of CSAI in patients with more severe ICDs should be specifically investigated in future studies under close monitoring.

Finally, we share the authors’ perspective that CSAI research must advance toward greater analytic precision, inclusion of at‐risk populations, and interdisciplinary approaches to ICD assessment and management—including non‐pharmacological strategies.

## Author Roles

At the end of the manuscript, list the itemized contributions in number/letter format, as below. These should include but are not restricted to: (1) Research project: A. Conception, B. Organization, C. Execution; (2) Statistical Analysis: A. Design, B. Execution, C. Review and Critique; (3) Manuscript Preparation: A. Writing of the first draft, B. Review and Critique.

C.D.: 1A, 1B, 1C, 2C, 3A, 3B.

P.A.D.B.: 1A, 1B, 2A, 2B, 2C, 3B.

M.F.: 1A, 1B, 1C, 2C, 3A, 3B.

## Disclosures

**Ethical Compliance Statement:** The authors confirm that the approval of an institutional review board was not required for this work. Informed patient consent was not necessary for this work. We confirm that we have read the Journal's position on issues involved in ethical publication and affirm that this work is consistent with those guidelines.

**Funding Sources and Conflict of Interest:** No specific funding was received for this work. The authors declare that there are no conflicts of interest relevant to this work.

**Financial Disclosures for the previous 12 months:** CD received Honoraria to speak from ORKYN, ASDIA, Homeperf and TEVA, consultancies from TEVA and ORION Pharma. PADB report no disclosures. MF received Honoraria to speak from AbbVie, ORKYN, and BIAL, consultancies from BIAL and LVL Medical; Grant from France Parkinson, HORIZON 2022 French Ministry of Health and MSA Coalition.

## Data Availability

Data available on request due to privacy/ethical restrictions.
